# *Pleurotus giganteus* (Berk. Karun & Hyde), the giant oyster mushroom inhibits NO production in LPS/H_2_O_2_ stimulated RAW 264.7 cells via STAT 3 and COX-2 pathways

**DOI:** 10.1186/s12906-016-1546-6

**Published:** 2017-01-13

**Authors:** Asweni Baskaran, Kek Heng Chua, Vikineswary Sabaratnam, Mani Ravishankar Ram, Umah Rani Kuppusamy

**Affiliations:** 1 Institute of Biological Sciences, Faculty of Science, University of Malaya, 50603 Kuala Lumpur, Malaysia; 2 Mushroom Research Centre, Faculty of Science, University of Malaya, 50603 Kuala Lumpur, Malaysia; 3 Department of Biomedical Science, Faculty of Medicine, University of Malaya, 50603 Kuala Lumpur, Malaysia

**Keywords:** Mushroom, *Pleurotus giganteus*, anti-inflammation, NO production, Lipopolysaccharide, Macrophage, STAT 3, COX-2

## Abstract

**Background:**

*Pleurotus giganteus* (Berk. Karunarathna and K.D. Hyde), has been used as a culinary mushroom and is known to have medicinal properties but its potential as an anti-inflammatory agent to mitigate inflammation triggered diseases is untapped. In this study, the molecular mechanism underlying the protective effect of ethanol extract of *P. giganteus* (EPG) against lipopolysaccharide (LPS) and combination of LPS and hydrogen peroxide (H_2_O_2_)-induced inflammation on RAW 264.7 macrophages was investigated.

**Method:**

The effect of EPG on nitric oxide (NO) production as an indicator of inflammation in RAW 264.7 macrophages was estimated based on Griess reaction that measures nitrite level. The expressions of inducible nitric oxide synthase (iNOS), cyclooxygenase-2 (COX-2), NF-kB activating protein (NKAP), signal transducer and activator of transcription 3 protein (STAT 3) and glutathione peroxidase (GPx) genes were assessed using real time reverse transcription polymerase chain reaction (RT-PCR) approach.

**Results:**

EPG (10 μg/ml) showed the highest reduction in the LPS-induced NO production in RAW 264.7 macrophages and significantly suppressed (*p* < 0.05) the expression iNOS, STAT 3 and COX-2. There was a significant increase (*p* < 0.05) in combination of LPS and H_2_O_2_- induced iNOS production when compared to the LPS-induced iNOS production in RAW 264.7 macrophages and this concurred with the NO production which was attenuated by EPG at 10 μg/ml. A significant (*p* < 0.05) down regulation was observed in the combination of LPS and H_2_O_2_-induced iNOS and GPx expression by EPG.

**Conclusions:**

Our data suggest that the anti-inflammatory activity of EPG is mediated via the suppression of the STAT 3 and COX-2 pathways and can serve as potential endogenous antioxidant stimulant.

## Background

Inflammation is the body’s network system that protects humans against environmental agents that are foreign to the body [1]. These harmful agents can be introduced through cuts, invasion of microorganisms, food, chemicals, or drugs. During inflammation, macrophages play a crucial role in counteracting the invasion of pathogens by releasing inflammatory mediators such as prostaglandin and pro-inflammatory cytokines (TNF-alpha, IL-6, IL-1α and IL-1β). These macrophages act upon the stimuli by a process called phagocytosis. During this process, phagocytes generate toxic products, which include reactive oxygen species such as nitric oxide (NO), hydrogen peroxide (H_2_O_2_) and superoxide anion (O_2_^-^) that contribute to oxidative stress at the cellular level. One of the potent stimuli for macrophages is the bacterial endotoxin (lipopolysaccharide), which activates the macrophages through the Toll-like receptors 4 (TLR 4) [2]. The process of recovering the cellular functions and structure back to its normal condition is called the acute phase of inflammation which is crucial to our body. Although these inflammatory mediators are essential in the defence mechanism, their overproduction causes chronic inflammation and the continuous destruction can be detrimental to tissues. Chronic inflammation has been linked to the pathogenesis of various diseases such as diabetes [3], Alzheimer’s [4], cardiovascular disease [5], and cancer [6] and these diseases have been shown to be the leading causes of death.

To date, non-steroidal anti-inflammatory drugs (NSAID’s) have been used as the treatment of choice for inflammatory conditions. However, the consumption of NSAID’s, has caused approximately 100,000 hospitalisations and 16,500 deaths due to gastrointestinal toxicity each year in the United States of America [7]. Besides gastrointestinal toxicity, Lacroix [8] reported that NSAID’s can also cause liver damage. These findings have motivated the search for anti-inflammatory treatments with fewer side effects.

Herbal products have established their importance as complementary therapies over the years but mushrooms have been gaining popularity for their therapeutic properties more recently [9]. Mushrooms have been well recognised as nutritional and functional foods and a source of potent bioactive compounds. In Malaysia, the genus *Pleurotus*, commonly referred to as ‘oyster mushrooms’, are cultivated. This ‘edible fungal food’ was discovered to have definite nutritive and medicinal values and has been popular in countries such as India, China and Japan. *Pleurotus giganteus* (Berk. Karunarathna and K.D. Hyde), formerly known as *Panus/Lentinus giganteus*, has been used as a culinary mushroom and is increasing in popularity for its medicinal properties and commercial prospects. Recently, it was reported that *P. giganteus* contained bioactive compounds that mimic neurite growth factor (NGF) which were accountable for neurite stimulation [10], possessed liver protection properties [11] and were a healthy dietary supplements for brain and cognitive health [12]. Hence, the aim of this study was to examine the anti-inflammatory effect of ethanol extract of *P. giganteus* (EPG) on LPS and combination of LPS and H_2_O_2_-induced RAW 264.7 macrophages. The effect of EPG on NO production and inducible nitric oxide synthase (iNOS), cyclooxygenase-2 (COX-2), NF-kB activating protein (NKAP), signal transducer and activator of transcription 3 protein (STAT 3) and glutathione peroxidase (GPx) expressions was investigated to unveil the mechanism of EPG as an anti-inflammatory action.

## Methods

### Mushroom species

The fresh basidiocarps of *P. giganteus* (commercial strain KLU-M 1227) were obtained from Nas Agro Farm, Sepang, Selangor, Malaysia and Dong Foong Manufacturing Sdn. Bhd., Kajang, Selangor, Malaysia. The mushroom identity (molecular fingerprinting) was authenticated by Dr. Tan Yee Shin from Mushroom Research Centre, University of Malaya. Voucher specimens were deposited in the Herbarium of University of Malaya (KLU-M 1227).

### Chemicals and reagents

Ethanol (99.8%) was purchased from System (Selangor, Malaysia), and dimethyl sulfoxide (DMSO) was purchased from Fisher Scientific Inc. (New Hampshire, USA). Dulbecco’s Modified Eagle’s Medium (DMEM), foetal bovine serum (FBS), *Escherichia coli* (O55:B5) lipopolysaccharide (LPS), N$\omega$-nitro-l-arginine-methyl ester (L-NAME), sulphanilamide, N-(1-naphty) ethylenediamine and phosphoric acid (H_3_PO_4_) were obtained Xfrom Sigma-Aldrich (St. Louis, MO, USA). 3-(4, 5-dimethylthiazol-2-yl)-2, 5-diphenyltetrazolium bromide (MTT) was purchased from Calbiochem, Merck Millipore (Darmstadt, Germany). Penicillin-streptomycin and fungizone were obtained from Biowest (MO, USA) and phosphate buffer saline (PBS) was purchased from Oxoid Ltd, Thermo Scientific (Hampshire, UK).

### Sample preparation

The basidiocarps of *P. giganteus* were sliced and freeze-dried for 48 h at -50 ± 2 °C. The freeze-dried basidiocarps were then powdered in a commercial Waring blender. The mushroom powder was kept in an airtight bottle under -20 °C prior to extraction.

### Preparation of ethanol extracts of P. giganteus (EPG)

The freeze-dried basidiocarp powder was soaked in a mixture of ethanol and water at a ratio of 8:2 (1 L) for three days at room temperature (25 °C). The solvent-containing extract was then decanted and filtered. The extraction process was repeated three times. The filtrates were then pooled, and the solvent was evaporated under reduced pressure using a rotary evaporator to yield a yellowish viscous extract [10].

### Cell culture

The murine macrophage cell line (RAW 264.7), catalogue number TIB-71, and was obtained from American Type Culture Collection, USA (ATCC). The cells were maintained in Dulbecco’s modified Eagle’s medium (DMEM, Sigma-Aldrich, St. Louis, MO, USA) supplemented with 10% heat-inactivated foetal bovine serum (FBS, Sigma-Aldrich, St. Louis, MO, USA), 100 units/mL of penicillin, 100 μg/mL of streptomycin, amphotericin B (Biowest, Nuaillé - France) and L–glutamine (Sigma-Aldrich, St. Louis, MO, USA) in a humidified atmosphere at 37 °C in 5% CO_2_. The medium was changed every 3-4 days as needed. The cells were cultured to reach at least 70% confluence prior to the assay [13].

### Cell viability

Cell viability was determined using the 3 [4, 5-dimethylthiazol-2-yl]-2, 5-diphenyltetrazolium bromide (MTT) assay. In the assay, yellow tetrazolium salt was reduced to insoluble purple formazan crystals by the mitochondrial dehydrogenases of viable cells. The RAW 264.7 macrophage cells (5 × 10^4^ cells per well) were seeded in a 96-well flat-bottomed culture plate and incubated at 37 °C overnight in a humidified environment of 5% CO_2_ and 95% air to allow cell adherence. Cells were then treated with 0.01–100 μg/mL of ethanolic extract of *P. giganteus* (EPG) for another 24 h. Subsequently, 5 mg/mL of MTT solution was added into each well and further incubated for 4 h to allow formazan crystal formation. The supernatant was then carefully removed, and 100 μl of dimethyl sulfoxide (DMSO, D5879, Sigma-Aldrich, St. Louis, MO, USA) was added into each well to dissolve the MTT formazan crystal. The absorbance was measured at 540 nm with a spectrophotometer (BioTek Instruments). The complete growth medium served as the blank, and cells incubated in medium only without mushroom extracts were used as the positive control [14].

### NO production

Nitrite that accumulated in the medium was measured as an indicator of NO production based on the Griess reaction. The RAW 264.7 cells were plated in a 96-well plate at a density of 4 × 10^5^ cells/well in 100 μL of culture medium for 24 h. Cells were then co-incubated with EPG (0.01–100 μg/mL) and 1 μg/mL of LPS for 24 h. After 24 h, the spent medium was collected for nitrite measurement. One-hundred microliters of the spent medium was transferred into a new 96-well plate and an equal amount of Griess reagent (0.1% N-1-[naphthyl] ethylenediamine-diHCl, 1% sulphanilamide and 5% H_3_PO_4_) was added to each well. The absorbance was measured at 550 nm using a microplate reader (BioTek Instruments) after a 15 min incubation period. The amount of NO was calculated using a sodium nitrite standard curve [14]. A strong iNOS inhibitor, NG-Nitro-L-Arginine Methyl Ester (L-NAME), was used as a positive control in this experiment. The units were expressed as nitrite in micromolar (μM).

### Real time: RT-PCR

The total RNA was isolated from the macrophage cells treated with EPG (10 μg/ml) using Ambion RNAqueous-Micro Kit (Applied Biosystems, USA). The purity of recovered total RNA was estimated by calculating the ratio of absorbance reading between 260 nm and 280 nm. The integrity of RNA was estimated using 2100 Bioanalyzer (Agilent Technologies, USA). Purified RNA with a $A$_260_/$A$_280_ ratio between 1.8 and 2.0 and RIN values between 8 and 10 were used to synthesize complementary DNA (cDNA) by the polymerase chain reaction (PCR) approach. A High Capacity cDNA Reverse Transcription Kit (Applied Biosystems, USA) which contained all reagents needed for reverse transcription (RT) of total RNA to single-stranded cDNA was used in this study. The mixture was then loaded into a thermal cycler (Eppendorf, USA), and PCR was carried out according to optimized thermal cycling conditions provided by the manufacturer. Table 1 shows the list of genes investigated in this study and the corresponding accession numbers. Endogenous control used in this study was eukaryotic beta actin with FAM/MGB probe. Dexamethasone and acetylsalicylic acid (aspirin) (a commercially available anti-inflammatory drug) were used as positive controls. The quantification approach used was the comparative CT method, also known as 2^-ΔΔCt^ method [15].

**Table 1:** The general abbreviation of genes selected for this study and corresponding assay ID and accession number were obtained from the Applied Biosystems website and NCBI database

No.	Gene name and abbreviation	Assay ID	Accession number
1	Nuclear factor-κB (NF-κB)	Mm 0482418_m1	NM_025937.4
2	Inducible nitric oxide synthase (iNOS)	Mm 0440502_m1	NM_010927.3
3	signal transducer and activator of transcription 3 protein (STAT 3)	Mm 1219775_m1	NM_011486.4
4	Glutathione peroxidase 3 (GPx 3)	Mm00492427_m1	NM_008161.2

Assay ID refers to the Applied Biosystems Gene Expression Assays inventoried kits with proprietary primer and TaqMan® probe mix. Assay ID with ‘Mm’ is referred to as ‘Mus musculus’. All Gene Expression Assay kits indicated are FAM/MGB probed

### Statistical analysis

The means of data were subjected to a one-way analysis of variance (ANOVA) and the significance of difference between means was determined by the Duncan’s multiple range tests. Values with *p* < 0.05 were regarded as statistically significant. The software used to determine the significance level was SPSS Statistics 17.0.

## Results and discussion

### Effects of EPG on cell viability

**Fig. 1:**
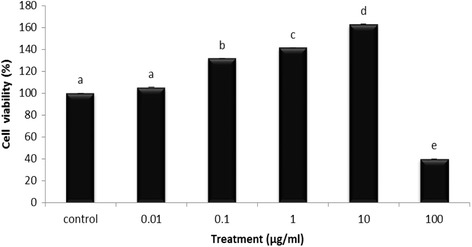
Effects of ethanol extracts of *P. giganteus* (EPG) studied at different concentration on RAW 264.7 macrophage’s cell viability. Control = negative control (untreated cells) taken as 100% viability. Each bar represents the mean cell viability defined as ratio of absorbance of treated to untreated cells (%) ± SD of three independent experiments carried out in triplicates. The different *alphabets* denote significant difference (*p* < 0.05) from the corresponding value of the negative control

Mushrooms are among the most prominent functional foods for humans. They are now known as the new generation of “bio therapeutics” [16] with antioxidant, anti-diabetic, anti-tumour, anti-hypertensive and anti-inflammatory properties. The Natural Health Products (NHPs) organization has shown interest, particularly in the prevention and treatment of several inflammation-caused chronic diseases, in using natural products. *Pleurotus giganteus* is a newly cultivated mushroom that is now being explored for its medicinal properties. Inflammation can be defined as the orchestrated response of inflammatory mediators such as pro-inflammatory cytokines (TNF-α, IL-6, IL-1), prostaglandin, and the free radical NO, against tissue injury or infection. Prior to the determination of the potential anti-inflammatory activity of EPG, the cytotoxic effect of EPG was assessed. Figure 1 shows the percentage of viable RAW 264.7 macrophages incubated with various concentrations of EPG compared to the untreated cells (negative control). A dose-dependent increase of cell viability was observed at the concentration range of 0.01–10 μg/ml of EPG tested. The maximum dose (100 μg/ml) tested, showed a significant (*p* < 0.05) decrease in cell viability. The cell viability for this particular concentration was only 39.9% as compared to the control. This could be due to the oversaturation of compounds in that particular concentration which led the cells to have a reduced viability. Hence, the concentration of EPG used was limited to a maximum of 10 μg/ml. Among all the concentration tested, 0.1, 1 and 10 μg/ml showed a significant (*p* < 0.05) increase in cell viability compared to the control. The EPG at 10 μg/ml showed the highest viability (162.98%). The lower concentrations did not affect the cell viability instead showed an increase viability of the RAW 264.7 macrophages. However, at higher concentration (100 μg/ml) the viability was significantly reduced. Similar increase in viability was also observed when EPG was tested with neurite cells (PC 12) [10]. These non-cytotoxic extracts were then further tested on LPS induced NO production in RAW 264.7 macrophage.

### Effects of EPG on NO production

**Fig. 2:**
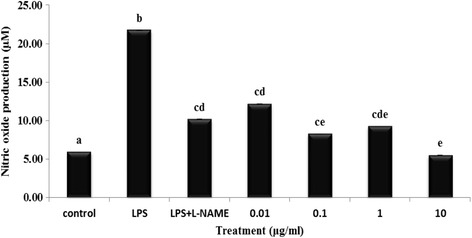
Effects of ethanol extracts of *P. giganteus* (EPG) studied at different concentrations on lipopolysaccharide (LPS) – induced nitric oxide (NO) production by RAW 264.7 macrophage cells. Control = negative control (untreated cells); L-NAME = L-NG-nitro arginine Methyl Ester (100 μM). Each *bar* represents the mean of NO production, defined as ratio of absorbance of treated to untreated cells (%) ± SD of three independent experiments carried out in triplicates. The different *alphabets* denote significant difference (*p* < 0.05) from the corresponding value for control

Although NO is responsible for the host defence mechanism [17], a high level of NO concentration can cause toxicity and damage to host cells. Excessive NO production is associated with pathogenesis of inflammatory diseases including atherosclerosis [18], vascular disease [19] and septic shock [20]. Bioactive compounds found in plants have been shown for years to inhibit inflammatory mediators. Lately, numerous mushrooms have already been proven to have anti-inflammatory activity by testing their inhibitory efficacy against the nitric oxide production [21]. Ethanol extract of *Russula virescens*, showed a reduction of the NO production in LPS-activated RAW 264.7 macrophages [22]. Besides, methanol extracts of edible mushrooms were also reported to inhibit the production of NO [21]. Nitric oxide is synthesized by iNOS and cyclooxygenase-2 (COX-2). Overexpression of both iNOS and COX-2 is commonly associated with inflammation. Therefore, the production of NO and the expression of iNOS and COX-2 can be an important target in the treatment or control of inflammation. Figure 2 illustrates the effect of EPG on NO production. The NO production measured as nitrite was increased significantly (*p* < 0.05) to 21.75 μM when RAW 264.7 cells were induced with 1 μg/ml of LPS, compared to 5.92 μM of the negative control without LPS. The various concentrations of EPG (0.01, 0.1, 1 and 10 μg/ml) showed significant (*p* < 0.05) reduction in LPS-induced NO production in RAW 264.7 cells (44.1%, 62.1%, 57.5% and 74.7% respectively). L-NAME (a strong iNOS inhibitor) caused a 63.5% reduction in the LPS-induced NO production in RAW 264.7 macrophages at a concentration of 100 μM while EPG at 10 μg/ml also showed significant inhibitory potency. Interestingly, ethanol extract of a very well-known mushroom *Ganoderma lucidum* exerted similar inhibitory effect on BV 2 microglial cells (a neuronal macrophage) but at a higher concentration (1 mg/mL) [23]. Hence, EPG (10 μg/ml) was used in the subsequent experiments to further confirm the presence of anti-inflammatory effects and unveil the possible mode of action on the RAW 264.7 macrophage cells using gene expression studies.

### Effect of EPG on the inflammatory genes

**Fig. 3:**
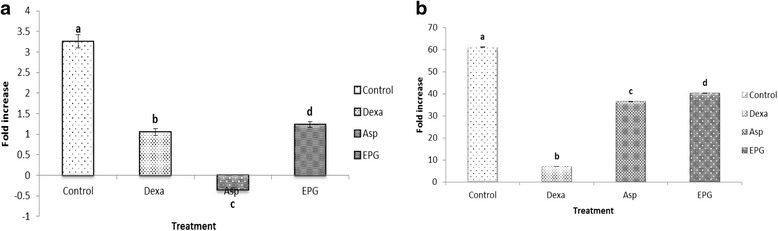
Effect of ethanol extract of *P. giganteus* (EPG) on the (**a**) iNOS and (**b**) COX-2 expression in lipopolysaccharide (LPS) stimulated RAW 264.7 macrophage cells. Results are expressed as fold of increase compared to LPS treatment as control. Fold increase values are calculated relative to the ACTB gene. Control = cell treated with LPS only; Dexa = dexamethasone; Asp = Aspirin; EPG = ethanol extract of *P. giganteus* at 10 μg/mL. Data expressed as mean ± S.D (*n* = 3). The different alphabets denote significant difference (*p* < 0.05) from the corresponding value for control

To elucidate whether the inhibitory effect observed on NO production (Fig. 2) by the extract was due to the down-regulation of the iNOS and COX-2 expression, gene expression study was conducted. Figure 3(a) shows the iNOS expression in LPS stimulated RAW 264.7 cells treated with 10 μg/ml of EPG. The expression of iNOS was significantly (*p* < 0.05) reduced by 2.65 fold in EPG treated cells when compared to the control. Dexamethasone and aspirin (commercially available anti-inflammatory drug) also showed a significant (*p* < 0.05) down regulation of the iNOS expression. Thus, it is feasible to suggest that the inhibitory effect of EPG on LPS- induced NO production was mediated by the inhibition of the iNOS expression. The level of expression of COX-2 in RAW 264.7 macrophages was also significantly (*p* < 0.05) inhibited by EPG (Fig. 3(b)), mimicking the inhibitory pathway used by aspirin and dexamethasone. It is well studied that NSAIDs use COX-2 pathway in inhibiting inflammation [24].

**Fig. 4:**
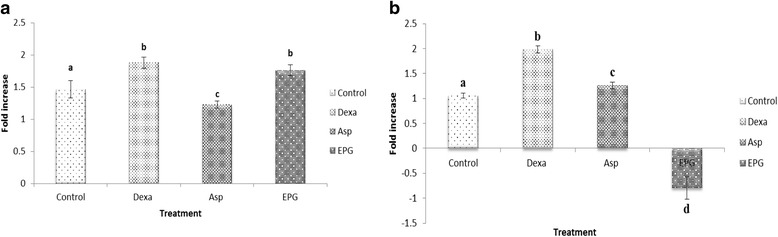
Effect of ethanol extract of *P. giganteus* (EPG) on the NF-kB (**a**) and STAT-3 (**b**) expression in lipopolysaccharide (LPS)-stimulated RAW 264.7 macrophage cells. Results are expressed as fold of increase compared to LPS treatment as control. Fold increase values are calculated relative to the ACTB gene. Control = cell treated with LPS only; Dexa = dexamethasone; Asp = Aspirin; EPG = ethanol extract of *P. giganteus* at 10 μg/mL. Data expressed as mean ± S.D (*n* = 3). The different *alphabets* denote significant difference (*p* < 0.05) from the corresponding value for control

It has been well documented that the expression of iNOS is regulated by the transcription factors NF-κB and STAT-3 and the exact pathway or mechanism remains unclear. The suppression of JAK2-STATs activation was reported to result in the inhibition of the LPS–induced production of NO and also the expression of iNOS [25], which is consistent with the anti-inflammatory properties of EPG demonstrated in this study. Therefore, the gene expression of NF-κB activating protein (NKAP) and STAT-3 was investigated in this study as iNOS is mainly produced via these transcriptional factors. Based on the data shown, EPG significantly reduced the expression of STAT-3 but not NKAP expression (Fig. 4). The expression of NKAP was unchanged (Fig. 4, panel a) whereas as indicated earlier, LPS-dependent induction of STAT-3 was markedly (*p* < 0.05) suppressed by EPG (Fig. 4, panel b). EPG also showed better reduction in the LPS-induced expression of STAT-3 (Fig. 4, panel b) in RAW 264.7 cells when compared to dexamethasone and aspirin. Therefore, it is pertinent to suggest that EPG exerts its anti-inflammatory activity by suppressing JAK2-STAT pathway. Phan reported that *P.giganteus* has high glycine (non-essential amino acid) content which acts on inflammatory cells to suppress activation of transcription factors and the formation of free radicals and inflammatory cytokine [26, 27]. Hence, this could cause the reduction of inflammatory mediators by EPG as mentioned above.

**Fig. 5:**
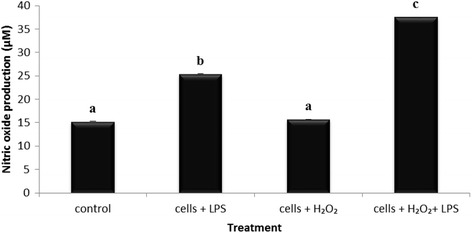
Effects of lipopolysaccharide (LPS), hydrogen peroxide (H_2_O_2_) and the combination effect of LPS and H_2_O_2_ on the production of NO by the RAW 264.7 macrophage cells. Untreated cells were used as the control. Data expressed as mean ± S.D (*n* = 3). The different *alphabets* denote significant difference (*p* < 0.05) from the corresponding value for control

Inflammation and oxidative stress are closely linked phenomenon. Oxidative stress occurs when there is an imbalance of reactive oxygen species (ROS) such as superoxide radical (O_2_^-^), hydrogen peroxide (H_2_O_2_) and hydroxyl radical (OH) and the antioxidant defences. This condition can cause damage to cell structure and function which leads to inflammation. Hseu reported that *Antrodia camphorata*, a new basidiomycete in the Polyporaceae (Aphyllophorales), attenuated the LPS-induced ROS production [28]. Therefore, in order to assess the protective effect of EPG against concurrent occurrence of inflammation and oxidative stress, the following experiments were carried out. Firstly, NO production was measured with the stimulation of H_2_O_2_ only, LPS only and the combination of LPS and H_2_O_2_ in RAW 264.7 cells. Figure 5 shows the differences in the production of NO using different stimulators. The NO production in RAW 264.7 cells treated with H_2_O_2_ only (0.125 μM) was comparable to the basal level which was similar to the finding reported by Eguchi using the BV-2 microglial cells [29]. The plausible explanation for this discrepancy is the absence of phagocytes which are usually triggered in the presence of pathogens or infection (eg. LPS), which in turn lead to the release of NO. However, LPS (1 μg/ml) induced NO production in the presence of H_2_O_2_ was significantly (*p* < 0.05) enhanced (37.6 μM) when compared to cells treated with only LPS (25.4 μM). This could be due to the ability of H_2_O_2_ to diffuse throughout the mitochondria and across cell membranes and act as a signal initiation molecule in enhancing NO production by LPS via the expression of IFNβ which inevitably leads to inflammation [30–32]. An overproduction of NO and accumulation of H_2_O_2_ will cause cellular damage and toxicity to the cell. Thus, there will be limited antioxidants to scavenge these oxidants and hence oxidative stress occurs. This study supports the insights into the co-relation between ROS-induced oxidative stress and inflammation.

**Fig. 6:**
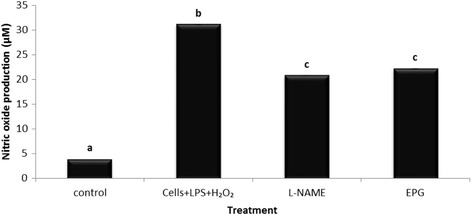
Effects of ethanol extracts of *P.giganteus* (EPG) by LPS (1 μg/ml) / H_2_O_2_ (0.125 μM) – induced nitric oxide (NO) production by RAW 264.7 macrophage cells. EPG = ethanol extract of *P.giganteus* (10 μg/ml); L-NAME = L-NG-Nitro arginine Methyl Ester (100 μM)*.* Data expressed as mean ± S.D (*n* = 3). The different *alphabets* denote a significant difference (*p* < 0.05) from the corresponding value for control

Miligan also reported that LPS-induced iNOS and NO production were enhanced in the presence of H_2_O_2_ and that H_2_O_2_ augmented the steady-state level of iNOS mRNA [32]. To study the effects of EPG on the combination effect of LPS and H_2_O_2_ on the production of NO by the RAW 264.7 macrophage cells, cells were primed with EPG at 10 μg/ml and co-incubated with LPS and H_2_O_2_ to induce the enhanced production of NO by the cells. There was a significant (*p* < 0.05) reduction of NO production (28.69%) by the RAW 264.7 cells when treated with EPG (Fig. 6) despite having a highly activated NO production. EPG showed comparable reduction with L-NAME (33.25%) in NO production.

**Fig. 7:**
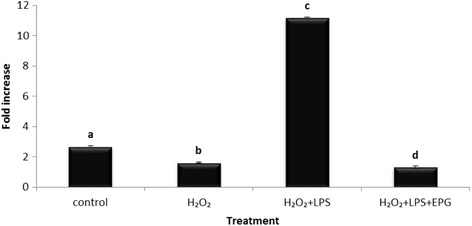
Effects of ethanol extract of *P. giganteus* on the iNOS expression in LPS, H_2_O_2_ and combination of LPS and H_2_O_2_ stimulated RAW 264.7 macrophage cells. Results are expressed as fold of increase compared to LPS treatment as control. Fold increase values are calculated relative to the ACTB gene. EPG = ethanol extract of *P. giganteus*. Data expressed as mean ± S.D (*n* = 3). The different *alphabets* denote significant difference (*p* < 0.05) from the corresponding value for control

Similarly the iNOS gene expression was tested to confirm the inhibitory effect of EPG on the RAW 264.7 macrophage cells. As shown in Fig. 7, EPG significantly (*p* < 0.05) down regulated (8.47 fold) the iNOS gene expression as compared to the LPS and H_2_O_2_-induced macrophage cells. The inhibition of iNOS expression was very high but the inhibition of NO production was not as great. We are unable to give an explanation based on our present data. Nevertheless, we postulate that the existing basal level iNOS (prior to the suppression of the gene) could contribute to the NO production to a certain extent. Generally, oyster mushrooms are well known to be potent anti-inflammatory agents [33] and the present study supports this view.

**Fig. 8:**
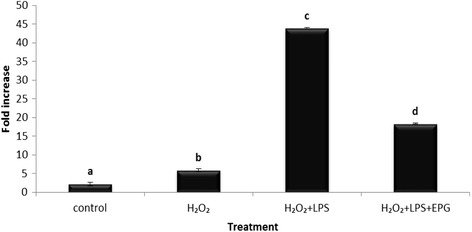
Effect of ethanol extract of *P. giganteus* on the GPx expression in LPS and H_2_O_2_-induced RAW 264.7 macrophage cells. Results are expressed as fold of increase compared to LPS treatment as control. Fold increase values are calculated relative to the ACTB gene. EPG = ethanol extract of *P. giganteus*. Data expressed as mean ± S.D (*n* = 3). The different *alphabets* denote a significant difference (*p* < 0.05) from the corresponding value for control

In order to further understand the mechanism of action in LPS and H_2_O_2_-induced inflammatory response, the GPx gene expression, which is the anti-oxidant enzyme responsible for the reduction of free H_2_O_2_ to water, was examined. The results in Fig. 8 show that the GPx expression level was higher when stimulated with LPS and H_2_O_2_ compared to LPS or H_2_O_2_ only. The presence of LPS could possibly increase the diffusibility of H_2_O_2_ across the cell membrane. This finding supports an earlier report which showed that the increase in GPx activity was a response againts increased availability of H_2_O_2_ in the cells [34]. EPG also showed a 2.4 fold reduction in GPx expression (Fig. 8). According to Phan, *P. giganteus* is a mushroom that has high phenolic content which positively correlates with its antioxidant activity (free radical scavenging and ferric reducing power) [26]. Lai also reported that the phenolic compounds from the pigeon pea (*Cajanus cajan* L.) correlates with the high antioxidant activity and possess anti-inflmmatory effect as well [35]. This could possibly explain the capability of EPG in inactivating H_2_O_2_ (a form of oxidant). These results enhance the understanding of the correlation between inflammation and oxidative stress.

## Conclusion

In summary, to the best of our knowledge, this study is the first to report on the anti-inflammatory activity of EPG by down-regulating LPS-induced NO production, iNOS, COX-2 and STAT-3 expression in RAW 264.7 cells. The EPG did not show any cytotoxicity on the RAW 264.7 cells up to a dose of 10 μg/ml. EPG was also effective in reducing the iNOS expression in H_2_O_2_ enhanced LPS-induced NO production in RAW 264.7 cells. The ethanol extract of *Pleurotus giganteus* has promising anti-inflammatory activity which acts via the suppression of the STAT 3 and COX-2 pathway and may be considered as a functional food.

## Abbreviations

COX-2: Cyclooxygenase-2
DMEM: Dulbecco’s Modified Eagle’s Medium
DMSO: Dimethyl sulfoxide
EPG: Ethanol extract of *P.giganteus*
FBS: Fetal bovine serum
GP_x_: Glutathione peroxidase JAK2-STATs
H_2_O_2_: Hydrogen peroxide
H_3_PO_4_: Sulphanilamide, N-(1-naphty) ethylenediamine and phosphoric acid
IFNβ: Interferon beta
IL-6: Interleukin-6
IL-α: Interleukin-alpha
IL-β: Interleukin-beta
iNOS: Inducible nitric oxide synthase
JAK-STAT: Janus kinase-STAT
L-NAME: N$\omega$-nitro-l-arginine-methyl ester
LPS: Lipopolysaccahride
MTT: 3[4, 5-dimethylthiazol-2-yl]-2, 5-diphenyltetrazolium bromide
NF-κβ: Nuclear factor-kappa beta
NGF: Nerve growth factor
NKAP: NF-κβ activating protein
NO: Nitric oxide
NSAID’s: Non-steroidal anti-inflammatory drugs
O_2_^-^: Superoxide anion
OH: Hydroxyl radical
PBS: Phosphate buffer saline
ROS: Reactive oxygen species
RT-PCR: Real time-polymerase chain reaction
STAT3: Signal transducer and activation of transcription 3 protein
TLR4: Toll-like receptor 4
TNF-α: Tumour necrosis factor alpha
